# Modified intraocular lens power selection method according to biometric subgroups Eom IOL power calculator

**DOI:** 10.1038/s41598-024-54346-9

**Published:** 2024-02-20

**Authors:** Youngsub Eom, So Hyeon Bae, Seul Ki Yang, Dong Hyun Kim, Jong Suk Song, David L. Cooke

**Affiliations:** 1 Department of Ophthalmology, Korea University Ansan Hospital, 123, Jeokgeum-ro, Danwon-gu, Ansan-si, Gyeonggi-do, 15355 South Korea; 2 Department of Ophthalmology, Korea University College of Medicine, Seoul, Republic of Korea; 3 Department of Ophthalmology, Emory University School of Medicine, Emory Clinic Building B, 1365B Clifton Road, Atlanta, NEGA 30322 USA; 4 Space Optics Laboratory, Department of Astronomy, Yonsei University, Seoul, Republic of Korea; 5 Satellite System 3 Team, Hanwha Systems Co., Ltd., Yongin‑si, Gyeonggi‑do, Republic of Korea; 6 Great Lakes Eye Care, 2848 Niles Road, Saint Joseph, MI 49085 USA; 7 Department of Neurology and Ophthalmology, School of Osteopathic Medicine, Michigan State University, East Lansing, MI USA

**Keywords:** Intraocular lens, Power, Biometry, Calculation, Formula, Medical research, Outcomes research

## Abstract

This study evaluates the accuracy of a newly developed intraocular lens (IOL) power calculation method that applies four different IOL power calculation formulas according to 768 biometric subgroups based on keratometry, anterior chamber depth, and axial length. This retrospective cross-sectional study was conducted in at Korea University Ansan Hospital. A total of 1600 eyes from 1600 patients who underwent phacoemulsification and a ZCB00 IOL in-the-bag implantation were divided into two datasets: a reference dataset (1200 eyes) and a validation dataset (400 eyes). Using the reference dataset and the results of previous studies, the Eom IOL power calculator was developed using 768 biometric subgroups. The median absolute errors (MedAEs) and IOL Formula Performance Indexes (FPIs) of the Barrett Universal II, Haigis, Hoffer Q, Holladay 1, Ladas Super, SRK/T, and Eom formulas using the 400-eye validation dataset were compared. The MedAE of the Eom formula (0.22 D) was significantly smaller than that of the other four formulas, except for the Barrett Universal II and Ladas Super formulas (0.24 D and 0.23 D, respectively). The IOL FPI of the Eom formula was 0.553, which ranked first, followed by the Ladas Super (0.474), Barrett Universal II (0.470), Holladay 1 (0.444), Hoffer Q (0.396), Haigis (0.392), and SRK/T (0.361) formulas. In conclusion, the Eom IOL power calculator developed in this study demonstrated similar or slightly better accuracy than the Barrett Universal II and Ladas Super formulas and was superior to the four traditional IOL power calculation formulas.

## Introduction

Improved postoperative refractive error and visual acuity can be achieved after cataract surgery with the accurate calculation of the implanted intraocular lens (IOL) power^1^. Various IOL power calculation formulas have recently emerged^2–5^. However, there are still prediction errors in IOL power calculation formulas^4,6^.

There are several causes of inaccurate IOL power calculation, such as biometry measurement error, effective lens position (ELP) prediction error in the IOL power calculation formula, and eyes with biometry that deviates from the average. Even if an optical biometer improves the accuracy and reproducibility of a measurement, errors due to the measurement method itself can occur. For example, in the corneal power (keratometry [K]) measurement, many devices only measure the anterior corneal curvature. An error can occur when the anterior/posterior corneal curvature ratio is outside the average range.^7^ In measuring the axial length (AL), errors often occur when measuring extreme axial lengths since most instruments use a one-segment regression formula (Traditional AL) for the intraocular medium.^8,9^ Traditional AL differs from the sum-of-segments AL in eyes with extreme ALs. Various methods have been proposed to increase the accuracy of the ELP prediction, and new formulas applying these methods have emerged^10–12^.

Eyes with a short or long AL are representative of eyes that deviate from the average biometry values and thus decrease the accuracy of IOL power calculations.^3,13,14^ Previous studies have emphasized that the accuracy of IOL power calculations is lower in eyes where AL, K, and anterior chamber depth (ACD) deviate from the average ranges^7,15–17^. In a previous study, we proposed a method to select the recommended IOL power calculation formula using 448 combinations of K-ACD-AL biometric subgroups according to their biometry values^6^. After publishing that study, we noted two concerns. First, an IOL power could not be selected when an eye had biometry values that did not fall into one of the 448 biometric subgroups. Second, the look-up tables from our previous study could be eliminated by incorporating the tables into the formula itself. The current IOL power calculator was developed to address these two concerns. We widened the range of the biometric subgroups to 768 and eliminated the look-up tables by modifying the IOL power calculation formula selection method used in the previous study. The purpose of this study was to evaluate the accuracy of this new IOL power calculation formula according to the IOL power calculation study protocol presented by Hoffer and Savini^18^.

## Results

Of the 1200 reference subjects, 695 (57.9%) were female, and 615 (51.3%) were left eyes. The mean age was 67.0 ± 11.1 years. Of the 400 subjects in the validation dataset, 234 (58.5%) were female, and 201 (50.3%) were left eyes. There was no significant difference in age, sex, laterality, preoperative K, ACD, and AL values measured via IOLMaster 500, and IOL power implanted into eyes between the reference and validation datasets (Table 1). Four hundred eyes in the validation set were assigned to 141 out of the 768 biometric subgroups in which the recommended IOL power calculation method was selected. Table 2 shows the distribution of the number and percentage of eyes according to the combination of K, ACD, and AL in the validation dataset.

**Table 1:** Clinical characteristics of reference and validation datasets in this study.

	Reference dataset (n = 1,200)	Validation dataset (n = 400)	*P* value†
Age, y	67.0 ± 11.1	67.3 ± 10.6	0.698
Sex, n (%)			
Male:Female	505 (42.1): 695 (57.9)	166 (41.5): 234 (58.5)	0.861‡
Laterality, n (%)			
Right:Left eye	585 (48.8): 615 (51.3)	199 (49.8): 201 (50.3)	0.773‡
Keratometry, D*	44.27 ± 1.60	44.22 ± 1.72	0.632
Anterior chamber depth, mm*	3.08 ± 0.47	3.10 ± 0.49	0.508
Axial length, mm*	23.57 ± 1.19	23.59 ± 1.25	0.796
IOL power, D	21.3 ± 2.8	21.2 ± 2.9	0.975

Data are reported as the mean (SD) except for sex and laterality, which are reported as n (%).

D, diopters; IOL, intraocular lens; SD, standard deviation.

* Keratometry, anterior chamber depth (from the corneal epithelium to the lens), and axial length measured by IOLMaster 500.

^†^Student’s *t*-test.

^‡^Chi-square test.

**Table 2:** Distribution of the number and percentage of eyes according to the combination of keratometry, anterior chamber depth, and axial length in the validation dataset (n = 400).

AL (mm)	K (D)	ACD (mm)			
		< 2.75	2.75–3.25	3.25–3.75	≥ 3.75
< 22.0 (n = 33)	< 42.0				
	42.0–44.0	2 (0.50)			
	44.0–46.0	9 (2.25)			
	≥ 46.0	13 (3.25)	9 (2.25)		
22.0–25.0 (n = 322)	< 42.0	9 (2.25)	8 (2.00)	6 (1.50)	
	42.0–44.0	21 (5.25)	53 (13.25)	42 (10.50)	6 (1.50)
	44.0–46.0	33 (8.25)	55 (13.75)	41 (10.25)	14 (3.50)
	≥ 46.0	5 (1.25)	20 (5.00)	6 (1.50)	3 (0.75)
25.0–26.0 (n = 28)	< 42.0		1 (0.25)	6 (1.50)	1 (0.25)
	42.0–44.0		2 (0.50)	6 (1.50)	4 (1.00)
	44.0–46.0		2 (0.50)	2 (0.50)	3 (0.75)
	≥ 46.0			1 (0.25)	
≥ 26.0 (n = 17)	< 42.0			3 (0.75)	2 (0.50)
	42.0–44.0	1 (0.25)			4 (1.00)
	44.0–46.0		3 (0.75)	2 (0.50)	2 (0.50)
	≥ 46.0				

Values are presented as a number (percentage).

AL = axial length; K = keratometry; D = diopters; ACD = anterior chamber depth.

AL, K, and ACD (from the corneal epithelium to the lens) are measured with IOLMaster 500.

For the validation set, the optimized A-constant for the Barrett Universal II formula was 119.32, and the optimized IOL constants a_0_, a_1_, and a_2_ for the Haigis formula were 1.306, 0.441, and 0.106, respectively. The optimized pACD for the Hoffer Q formula was 5.763, the optimized surgeon factor for the Holladay 1 formula was 1.967, the optimized A-constant for the Ladas Super formula was 119.20, the optimized A-constant for the SRK/T formula was 119.18, and the optimized A-constant for the Eom IOL power calculator was 119.24.

**Figure 1:**
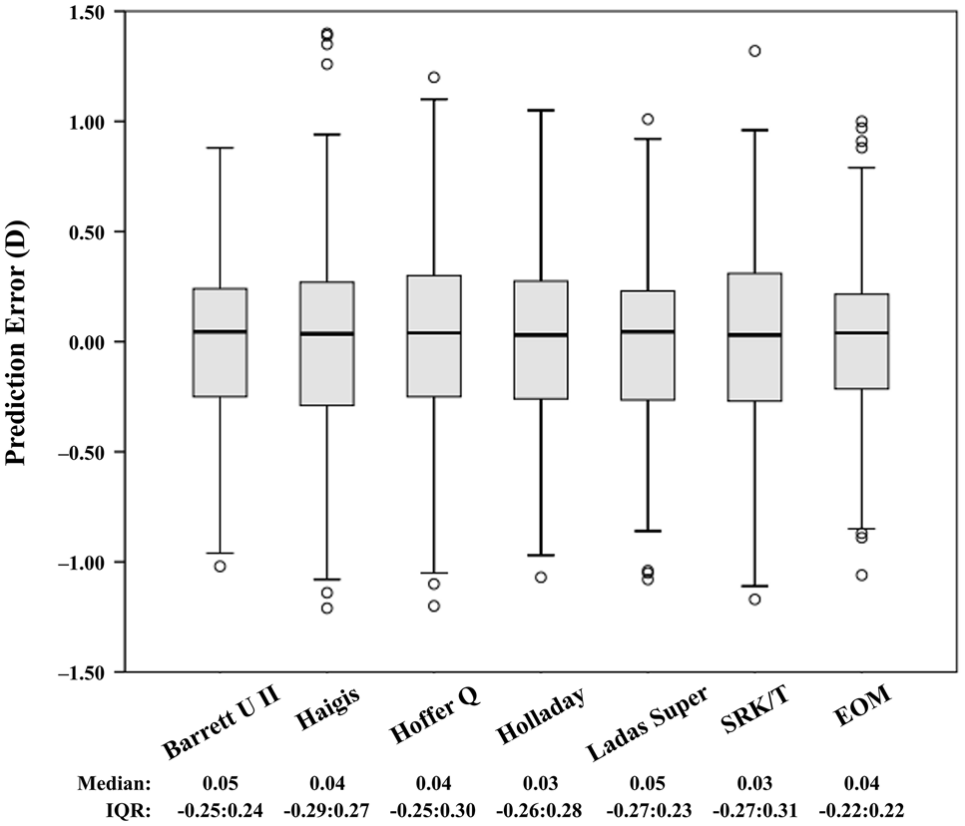
Box plot of prediction error determined using the Barrett Universal II, Haigis, Hoffer Q, Holladay 1, Ladas Super, SRK/T, and Eom formulas. IQR = interquartile range.

Figure 1 shows the box plots and distributions of prediction errors determined using the seven formulas. The Eom formula had the smallest interquartile range and the formulas with the least outliers in prediction error were the Barrett Universal II, Ladas Super, and Eom formulas. The MedAE values (interquartile range) of the Barrett Universal II, Haigis, Hoffer Q, Holladay 1, Ladas Super, SRK/T, and Eom formula were 0.24 (0.09:0.45), 0.27 (0.14:0.45), 0.30 (0.15:0.50), 0.27 (0.12:0.47), 0.23 (0.10:0.46), 0.28 (0.13:0.46), and 0.22 (0.09:0.42) D, respectively. The MAE values (± standard deviation) were 0.29 ± 0.23, 0.33 ± 0.26, 0.35 ± 0.26, 0.31 ± 0.23, 0.30 ± 0.24, 0.33 ± 0.25, and 0.28 ± 0.23 D, respectively. The MedAE of the Eom formula (0.22 D) was significantly smaller than that of the other four formulas except for the Barrett Universal II (0.24 D) and Ladas Super formulas (0.23 D) (Friedman’s test with post hoc test; all *P* < 0.05). The percentage of eyes with a prediction error within ± 0.50 D was 81.5%, 79.3%, 76.5%, 78.5%, 79.3%, 79.8%, and 83.5% with the Barrett Universal II, Haigis, Hoffer Q, Holladay 1, Ladas Super, SRK/T and Eom formulas, respectively. The Cochran’s Q test indicated that the best results in the percentage of eyes with a prediction error within ± 0.50 D were obtained using the Eom and Barrett Universal II formulas (*P* = 0.006) (Table 3). The IOL FPI of the Eom formula was 0.553, which ranked first, and that of the Ladas Super formula was 0.474, which ranked second, followed by the Barrett Universal II (0.470), Holladay 1 (0.444), Hoffer Q (0.396), Haigis (0.392), and SRK/T formulas (0.361) (Table 3).

**Table 3:** Median absolute error, mean absolute error, mean prediction error, and intraocular lens Formula Performance Index calculated by the Barrett Universal II, Haigis, Hoffer Q, Holladay 1, Ladas Super, SRK/T, and Eom formulas (n = 400).

	Barrett U II	Haigis	Hoffer Q	Holladay 1	Ladas super	SRK/T	Eom	*P* value
MedAE, D (median)	0.24	0.27	0.30	0.27	0.23	0.28	0.22	< 0.001†
MAE, D (mean ± SD)	0.29 ± 0.23	0.33 ± 0.26	0.35 ± 0.26	0.31 ± 0.23	0.30 ± 0.24	0.33 ± 0.25	0.28 ± 0.23	
PE, D (mean ± SD)	0.00 ± 0.37	0.00 ± 0.42	0.00 ± 0.43	0.00 ± 0.39	0.00 ± 0.38	0.00 ± 0.41	0.00 ± 0.36	
± 0.25D, n (%)	210 (52.5)	181 (45.3)	189 (47.3)	188 (47.0)	210 (52.5)	186 (46.5)	217 (54.3)	
± 0.50D, n (%)	326 (81.5)	317 (79.3)	306 (76.5)	314 (78.5)	315 (78.8)	319 (79.8)	334 (83.5)	0.006‡
± 0.75D, n (%)	380 (95.0)	367 (91.8)	372 (93.0)	380 (95.0)	377 (94.3)	368 (92.5)	381 (95.3)	
± 1.00D, n (%)	399 (99.8)	392 (98.0)	392 (98.0)	392 (99.5)	396 (99.0)	395 (98.8)	399 (99.8)	
Slope*	0.029	-0.060	0.049	0.032	0.023	0.083	0.003	
FPI	0.470	0.392	0.396	0.444	0.474	0.361	0.553	

FPI, formula performance index; MAE, mean absolute error; MedAE, median absolute error; PE, prediction error; SD, standard deviation.

* Slope of the correlation between the prediction error and axial length.

^†^ Friedman test with post hoc test.

^‡^ Cochran’s Q test.

**Figure 2:**
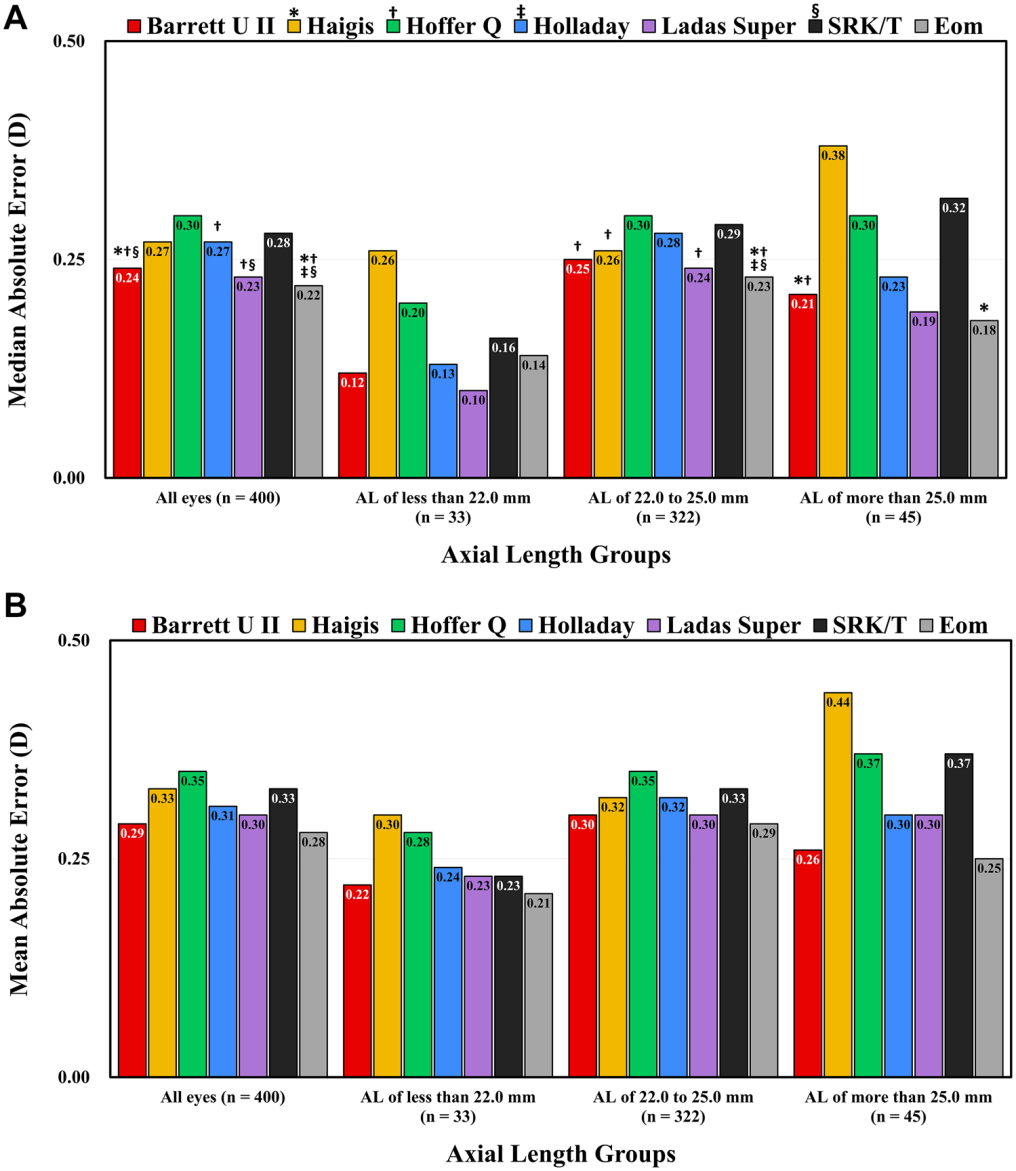
The median absolute error (**A**) and mean absolute error (**B**) of the axial length groups, calculated by the Barrett Universal II, Haigis, Hoffer Q, Holladay 1, Ladas Super, SRK/T, and Eom formulas. An asterisk (*) indicates a *P* value < 0.05 compared with the Haigis formula, a dagger (†) indicates *P* value < 0.05 compared with the Hoffer Q formula, a double dagger (‡) indicates a *P* value < 0.05 compared with the Holladay 1 formula, and a section mark (§) indicates a *P* value < 0.05 compared with the SRK/T formula by Friedman’s test after a post hoc test.

Figure 2 shows the MedAE and MAE values determined using the seven formulas for all eyes and subgroups grouped by AL. In eyes with an AL of less than 22.0 mm (n = 33), the MedAE of the Haigis formula (0.26 D) was the largest among the seven formulas (Friedman’s test; *P* = 0.007). However, there was no significant difference in the MedAE between formulas after a post hoc test. In eyes with an AL of between 22.0 mm and 25.0 mm (n = 322), the MedAE of the Eom formula (0.23 D) was significantly smaller than that of the other formulas except for the Barrett Universal II (0.25 D) and Ladas Super formulas (0.24 D). The MedAE of the Barrett Universal II (0.25 D), Haigis (0.26 D), and Ladas Super formulas (0.24 D) were significantly smaller than that of the Hoffer Q formula (0.30 D). In eyes with an AL greater than 25.0 mm (n = 45), the MedAE of the Barrett Universal II formula (0.21 D) was smaller than those of the Haigis (0.38 D) and Hoffer Q formulas (0.30 D). The MedAE of the Eom formula (0.18 D) was significantly smaller than that of the Haigis formula.

**Figure 3:**
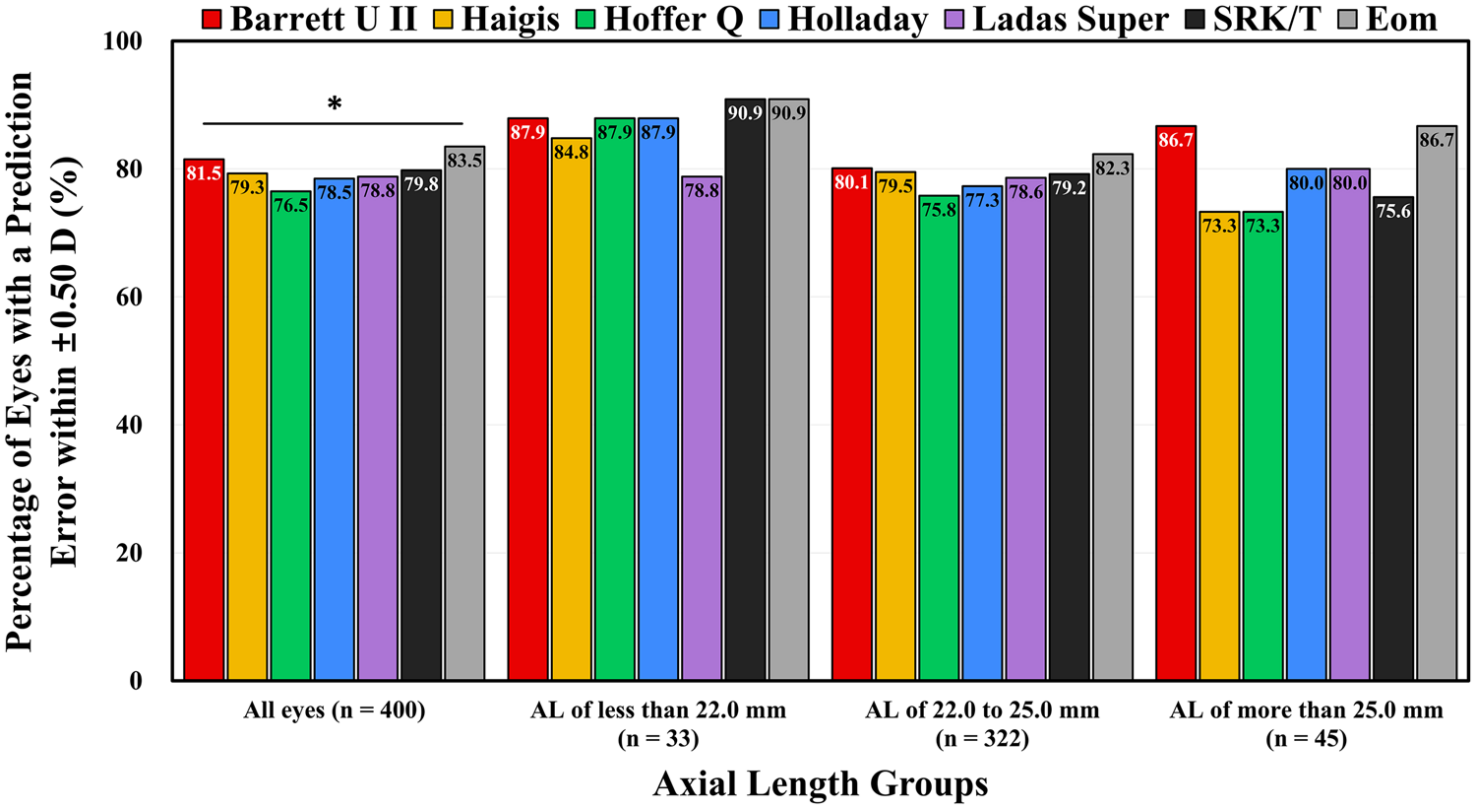
The percentage of eyes with a prediction error within ± 0.50 D in the axial length groups determined by the Barrett Universal II, Haigis, Hoffer Q, Holladay 1, Ladas Super, SRK/T, and Eom formulas. An asterisk (*) indicates a P value < 0.05 compared among the seven formulas using a Cochran’s Q test.

In all eyes, the best results in the percentage of eyes with a prediction error within ± 0.50 D were obtained using the Eom and Barrett Universal II formulas (*P* = 0.006) (Fig. 3). In eyes with an AL of between 22.0 and 25.0 mm, the Eom formula tended to have the greatest percentage of eyes with a prediction error within ± 0.50 D among the seven formulas (*P* = 0.051). However, in eyes with an AL < 22.0 mm or > 25.0 mm, there were no significant differences in the percentage of eyes with a prediction error within ± 0.50 D among the seven formulas.

## Discussion

This study developed a new IOL power calculation method that utilizes an optimized tabular calculation method to select the optimal IOL power calculation formula among four formulas in 768 biometric subgroups. This study showed that the new IOL power calculator showed similar or slightly better accuracy in the refractive error prediction than the Barrett Universal II and Ladas Super formula and better accuracy than the Haigis, Hoffer Q, Holladay 1, and SRK/T formulas. In addition, according to the FPI introduced by Wolfgang Haigis, the new IOL power calculator performed the best among the seven IOL power calculation formulas. The IOL power calculator derived in this study is similar to the method of selecting the recommended IOL power calculation formula in a previous study^6^, but there are three main differences. First, the CMAL, proposed by David L. Cooke, and the Wang-Koch Holladay polynomial non-linear regression equation were applied to adjust the AL to improve the accuracy of the IOL power calculation in the existing formulas^8^. Second, this study extended the range of biometric subgroups to allow the calculation of IOL power in most eyes. Third, there is no need to reference the IOL power calculation formula selection tables. Instead, the IOL power and predicted refraction values are calculated once the biometry values are entered into an online calculator or an Android application calculator, making this method easy to use.

Traditional optical biometers use a single refractive index for measuring the AL, and this could be a reason for unexpected refractive errors after cataract extractions^8,9,19^. Although an optical biometer using a segmental refractive index technology (Argos; Alcon, Fort Worth, TX, USA) has been developed, many clinics employ optical biometers using a single-segment regression formula, including the IOLMaster 700 (Carl Zeiss Medtec), which is considered the gold standard^20^. Cooke et al. introduced the method of calculating the sum-of-segments AL (CMAL) to improve the skewed prediction error in short and long eyes caused by optical biometers that do not measure individual ocular segment lengths. A previous study showed that the CMAL formula easily calculates the sum-of-segment AL using the traditional AL and improves the accuracy of the refractive error prediction for several IOL power calculation formulas in short and long eyes^8,21,22^. Until now, LT has been required for calculating CMAL. In this study, we used a version of CMAL which does not require LT. In another approach, Wang and Koch et al. introduced the Wang-Koch AL adjustment method to solve the hyperopic shift that occurs in long eyes^23^. Afterwards, the Wang-Koch Holladay polynomial non-linear regression equation, an improvement from the previous AL adjustment method, was published^24,25^. In the development of the new IOL power calculator, this study applied the CMAL in the Hoffer Q formula for all eyes and the Holladay 1 formula for short or medium eyes, the Wang-Koch AL adjustment in the Haigis formula for long eyes, and the Wang-Koch Holladay polynomial non-linear regression equation in the Holladay 1 formula for long eyes. Adjusting the method by applying the CMAL and Wang-Koch AL adjustment methods differently according to the formula allowed us to obtain all the advantages of each AL method. In addition, the SRK/T formula was used to gain the advantage of the original formula without AL adjustment.

Many IOL power calculation formulas, including calculators using artificial intelligence, have recently been introduced. The Hill-RBF calculator is the IOL power selection method that uses pattern recognition by artificial intelligence^26^. The Ladas Super formula is a method of using different IOL power calculation formulas depending on K and AL. This formula was improved by applying artificial intelligence (https://www.iolcalc.com/home; Accessed 7 Dec 2023)^27^. Although the IOL power calculator presented in this study does not use artificial intelligence, it is similar to artificial intelligence as it selects the optimal IOL power calculation formula by comparing four IOL calculation methods in each of the 768 biometric subgroups. In addition, the Eom IOL power calculator’s method do not end with a fixed algorithm. The algorithm and the IOL power calculation accuracy could be improved by gathering and refining reference data from published studies.

An appropriate research design and analysis method are important to compare the accuracy of the IOL power calculation formulas. In this regard, Hoffer et al. introduced a protocol for studies comparing IOL power calculation formulas in 2015 and updated the protocol in 2021^18,28^. This study investigated the accuracy of the Eom IOL power calculator by following the protocol proposed by Hoffer et al. as much as possible. Our new IOL power calculator was superior to the traditional IOL power calculation formulas and had a similar or slightly better accuracy than the Barrett Universal II and Ladas Super formulas.

There are some limitations to this study. Although we used 400 eyes to validate the study validation, there were few short or long eyes in the analysis. Second, of the 768 biometric subgroups, we included 141 subgroups in the validation dataset. Although this reduction in the number of subgroups was unavoidable since there are few eyes with long AL, the accuracy of IOL power calculation formulas cannot be assessed for special biometric subgroups without cases. The newly developed IOL power calculator demonstrated better accuracy than the other formulas for all eyes. However, although the new IOL power calculator had a higher percentage of eyes with a prediction error within ± 0.50 D than the other formulas in the subgroup analyses according to AL values (22.0 mm and 25.0 mm), there was no statistically significant difference among the seven formulas for short or long eyes. Thus, the new IOL power calculator accuracy must be verified in eyes with extreme biometry through research on eyes that deviate considerably from the average biometry values. A final limitation of this study is that it does not include LT. CMAL requires LT, but we used a modified CMAL, which is published here for the first time.

## Conclusion

The Eom IOL power calculator, which applies four different IOL power calculation formulas according to the biometry subgroup combined with K, ACD, and AL, showed superior accuracy compared with the Haigis, Hoffer Q, Holladay I, and SRK/T formulas and similar or slightly better accuracy than the Barrett Universal II and Ladas Super formulas. Surgeons can conveniently use the Eom IOL power calculator through an online calculator or an Android application calculator.

## Methods

### Study population

A total of 1600 eyes from 1600 patients who underwent phacoemulsification and a Tecnis ZCB00 (Johnson & Johnson Vision Care, Inc.) IOL in-the-bag implantation at our institute between August 2011 and January 2022 were enrolled in this study. Only eyes with a best-corrected visual acuity (BCVA) of 20/40 or better were included. The exclusion criteria were as follows: (1) a history of previous ocular surgery, such as corneal transplant or refractive surgery; (2) traumatic cataracts; (3) intraoperative complications, such as posterior capsule rupture; and (4) incomplete biometry measurements with an optical biometer. If both eyes met the inclusion criteria, the first eye operated on was selected. Institutional review board approval was obtained from the Korea University Ansan Hospital in Gyeonggi, Korea, for this study (2021AS0078). Since data were evaluated retrospectively, pseudonymously and were solely obtained to calculate the IOL power, the Ethics Committee of the Korea University Ansan Hospital waived the informed consent requirement. All research and data collection methods adhered to the tenets of the Declaration of Helsinki.

### Patient examination

Preoperative biometry, including K, ACD (from the corneal epithelium to the lens)^18^, and AL were measured using an IOLMaster 500 (Carl Zeiss Meditec, Jena, Germany). The IOL power was calculated with the Eom IOL power calculator, which uses an optimized tabular calculation method using four IOL power calculation methods: (1) the Haigis formula^29^ without (for eyes with an AL < 25.2 mm) or with Wang Koch adjustment (for eyes with an AL ≥ 25.2 mm)^23^; (2) the Hoffer Q formula^30–33^ using Cooke-modified AL (CMAL) for all AL values^8^; (3) the Holladay 1 formula^34^ using CMAL (for eyes with an AL < 24.0 mm)^8^ or the Wang-Koch Holladay polynomial non-linear regression equation (for eyes with an AL ≥ 24.0 mm)^24,25^; and (4) the SRK/T formula^35^.

The formula for CMAL has been previously published and requires lens thickness (LT).^8^ An unpublished version of CMAL for IOLMaster 500 was simultaneously developed without using LT. This unpublished version was used in this study as follows:

$$CMAL=3.574453886278630+0.610250243334448\times AL+0.014682933220208\times {AL}^{2}-0.000203380571583\times {AL}^{3}+0.038468287096921\times ACD$$

To use one SRK/T A-constant in the IOL power calculation formula as the input value of the IOL constant, the following constants of the Haigis, Hoffer Q, and Holladay formulas were used as the converted values: a_0_, a_1_, and a_2_ for the Haigis formula were calculated as the A-constant × 0.662−77.472, 0.400, and 0.100, respectively; the pACD for the Hoffer Q formula was calculated as the A-constant × 0.639−70.540; the surgeon factor for the Holladay 1 formula was calculated as the A-constant × 0.618−71.800. When the SRK/T A-constant was 119.3, the IOL constants a_0_, a_1_, and a_2_ for the Haigis formula were 1.505, 0.400, and 0.100, respectively, and the pACD for the Hoffer Q formula was 5.693, and the surgeon factor for the Holladay 1 formula was 1.927. The postoperative manifest refraction and BCVA at 4 m were measured at postoperative visits between 4 and 10 weeks. The measured refractions at 4 m were converted to 6 m by subtracting 0.08 D from the spherical equivalent^36^.

### Surgical technique

All phacoemulsification and IOL implantations were performed under topical anesthesia using 0.5% proparacaine hydrochloride (Paracaine; Hanmi Pharm, Seoul, Korea or Alcaine; Alcon Laboratories Inc, Fort Worth, TX, USA) by eleven different experienced surgeons at our institute. A 2.75-mm clear corneal incision and a continuous curvilinear capsulorrhexis were made. A standard phacoemulsification technique was performed using Stellaris (Bausch & Lomb, Rochester, NY, USA), and the IOL was implanted into the capsular bag using an injector system.

### EOM IOL power selection method

The included 1600 eyes from 1600 Korean patients were divided into two groups: (1) a reference dataset (n = 1200) for developing the formula and (2) a validation dataset (n = 400) for evaluating the accuracy of the developed formula. The reference dataset was divided into 768 biometric subgroups: AL (original AL measured with IOLMaster 500) from 18.75 mm to 30.75 mm in 0.5-mm steps; K from 40.0 D to 48.0 D in 1.0 D steps; and ACD from 2.25 mm to 4.25 mm in 0.5-mm steps. The postoperative spherical equivalent refraction of the subgroups from the 1200 eyes was used as a reference dataset. To account for missing data in the biometric subgroups, the sum of the predicted refraction and the prediction errors of the Haigis, Barrett universal II, Kane, and EVO 2.0 formulas, from the results of previous studies, in each biometry subgroup were obtained^3,4,37^. For the Kane and EVO 2.0 formulas, the effects of K and ACD were not reflected in the biometric subgroups because only refractive error data according to AL were available in previous studies^4,37^. Therefore, the sum of the predicted refraction and the prediction error of the Kane and EVO 2.0 formulas according to AL was equally applied in the biometry subgroup with the same AL even though K and ACD were different. For the Haigis and Barrett Universal II formulas, the sum of the mean prediction errors for K, ACD, and AL was added to the predicted refraction of each formula in each biometry subgroup. When the prediction errors for K, ACD, and AL had the same sign, the larger prediction error was selected for the sum of the mean prediction errors^6^. The average value of the sum of the predicted refraction and the prediction errors of four IOL calculation formulas in the biometry subgroup was used as a reference dataset.

The postoperative spherical equivalent refraction in the reference dataset and the predicted refraction were compared using four formulas (the Haigis formula with Wang-Koch adjustment, the Hoffer Q formula using CMAL, the Holladay 1 formula using CMAL or Wang-Koch Holladay polynomial non-linear regression equation, and the SRK/T formula). The formula with the smallest difference in predicted spherical equivalent refraction using the reference dataset was selected as the IOL power calculation formula to be used in the biometric subgroups of the Eom IOL power selection method^6^. If there was a difference of 0.2 D or more between the postoperative refraction in the reference dataset and the predicted refraction of the formula with the smallest difference, an offset value was added to the predicted refraction of that formula.

### Main outcome measures

The prediction error was defined as the difference between the postoperatively measured and the preoperative formula-predicted spherical equivalent refraction (postoperative manifest spherical equivalent—the preoperative predicted spherical equivalent refraction). The mean absolute error (MAE) and median absolute error (MedAE) were defined as the mean absolute value and the median absolute value of the prediction error, respectively. The percentage of eyes with a prediction error within ± 0.25 D, ± 0.50 D, ± 0.75 D, and ± 1.00 D were measured.

IOL constant optimization was conducted in each IOL power calculation formula leading to a zero mean prediction error in the validation dataset before investigating the accuracy of the IOL power calculation methods^18^. To compare the prediction accuracy among the IOL power calculation methods (Barrett Universal II, Haigis^29^, Hoffer Q^30–33^, Holladay 1^34^, Ladas Super^27^, SRK/T formula^35^, and Eom formulas) in the validation dataset (400 eyes), the MAE, MedAE, and percentage of eyes with a prediction error within ± 0.50 D were compared. In addition, those values were also compared among the IOL power calculation methods in subgroups according to AL values (22.0 mm and 25.0 mm).

To rank formula accuracy, the IOL Formula Performance Index (FPI) was compared between the IOL power calculation methods after calculating the FPI of each formula as follows^18^:

$$FPI= \frac{1}{SD+MedAE+10\times abs\left(m\right)+10\times {(n10)}^{-1}}$$

where *FPI* is the IOL Formula Performance Index, *SD* is the standard deviation of the prediction error, *MedAE* is the median absolute value of the prediction error, *abs(m)* is the absolute value of *m*, *m* is the slope of the correlation between the prediction error and the AL, and *n* is the inverse value of the percentage of eyes with a prediction error within ± 0.50 D.^18^.

### EOM intraocular lens power calculator

Excel (Microsoft, Inc., Redmond, WA, USA) was used to develop the Eom IOL power calculator. The generated excel calculator was converted to html format using MATLAB (MathWorks, Natick, MA, USA) to be used as an online calculator (http://www.eomiolcalc.com). An IOL power calculator application for Android was also built based on the generated excel calculator (available at the Google Play store).

### Statistical analysis

Descriptive statistics for all patient data were obtained using statistical software (Statistical Package for Social Sciences Statistics Standard 20; IBM Corp., Armonk, NY, USA). Student’s t-test and Chi-square test were used to compare parameters between the reference dataset and the validation dataset. Comparisons of the MedAE values among the IOL power calculation formulas were conducted using a Friedman repeated-measures test with a Dunn’s pairwise post hoc test. A Cochran’s Q test was performed to compare the percentage of eyes with a prediction error within ± 0.50 D among the IOL power calculation formulas. A linear regression analysis was performed to calculate the slope of the correlation between the prediction error and the AL. The results were considered statistically significant if the P value was less than 0.05.

## Data Availability

The datasets used and/or analysed during the current study available from the corresponding author on reasonable request.
